# Immune Cytolytic Activity for Comprehensive Insights of the Immune Landscape in Endometrial Carcinoma

**DOI:** 10.1155/2022/9060243

**Published:** 2022-07-18

**Authors:** Qiang Chen, Chongyang Wang, Xinyi Lei, Ting Huang, Renyu Zhou, Yuanzhi Lu

**Affiliations:** ^1^Department of Oncology, Cancer Diagnosis and Therapy Research Center, The First Affiliated Hospital, Jinan University, Guangzhou, Guangdong 510632, China; ^2^The First Affiliated Hospital, Jinan University, Guangzhou, Guangdong 510632, China; ^3^Biomedical Translational Research Institute, Faculty of Medical Science, Jinan University, Guangzhou, Guangdong 510632, China; ^4^Department of Gastric Surgery, Institute of Cancer Research and Basic Medical Sciences of Chinese Academy of Sciences, Cancer Hospital of University of Chinese Academy of Sciences, Zhejiang Cancer Hospital, Hangzhou, Zhejiang 310022, China; ^5^Department of Clinical Pathology, The First Affiliated Hospital, Jinan University, Guangzhou, Guangdong 510632, China

## Abstract

Immune checkpoint blockade (ICB) has been explored as a therapeutic strategy to recover the antitumor immune activities against endometrial cancer (EC) escaping from immune surveillance. Increasing evidence has indicated that microsatellite instability (MSI) is a promising biomarker to stratify patients for the ICB therapy. However, even in patients with MSI-High (MSI-H) endometrial cancers, PD-L1 inhibitors, avelumab, and durvalumab have shown only 27% of response rates. Therefore, there is an urgent need to discover new biomarkers for a predictive response to ICB therapy. In this study, we demonstrated that the immune cytolytic activity (CYT) index was significantly correlated with the development and response to immunotherapy in EC. The data showed that higher CYT was significantly associated with better clinical outcome, more antitumor infiltrating immune cells, fewer somatic copy number alterations, but a higher TMB (Tumor mutational burden) status. Furthermore, CYT-high EC was notably relevant to the high expression of various immune checkpoint molecules and showed more effective responses to ICB treatment. Taken together, this study provided new insights into the connection between diverse genetic events and the immune microenvironment in EC and indicated that the CYT status might be a promising biomarker to stratify patients with EC for ICB therapy.

## 1. Introduction

Endometrial cancer is a malignant gynecological tumor and the main treatment regimens included surgery, radiotherapy, chemotherapy, and endocrine therapy [1]. Patients with early-stage endometrial cancer usually benefit from surgery with the most favorable outcome, while advanced-stage patients who have metastasized or recurred lesions commonly tend to be refractory and have poor prognosis. In recent years, significant breakthroughs made on cancer immunotherapy, particularly great success in using immune checkpoint inhibitors alone or in combination with other therapeutic regimens, have been introduced in the clinical guidelines for treatment and management of various cancers including endometrial cancer [2].

In 2013, The *Cancer* Genome Atlas of America (TCGA) conducted a genomic, transcriptomic, and proteomic study of 373 endometrial cancer samples and proposed to distinguish four molecular subtypes of endometrial cancer [3]: POLE ultramutated (POLE), microsatellite instability (MSI) hypermutated, copy-number low (CN-Low), and copy-number high (CN High). Of these, tumors with POLE mutants and MSI phenotypes produce a large number of tumor-specific neoantigens and tumor-infiltrating lymphocytes (TILs) involved in the active immune microenvironment, resulting in the overexpression of PD-1 and PD-L1 [4]. This suggests that a more active immune response exists in the local tumor microenvironment of cancers with MSI+ and POLE hypermutant phenotypes, blocking PD-1/PD-L1; therefore, inducing an effective antitumor immune response, and that patients with MSI and POLE hypermutant phenotypes, may be a beneficial population for PD-1/PD-L1 inhibitors.

Despite this, immunotherapy appears to be limited in gynecological oncology, as some tumors exhibit relatively poor efficacy and low response ratio [5]. Therefore, one strategy to improve the prognosis of EC patients is to shift from a traditional bifurcation-based treatment model to a molecular-based precision therapy, using biomarkers to differentiate sensitive patients and predict treatment response. In other tumors (e.g., colorectal cancer), there is sufficient evidence to support the use of PD-L1 or PD-1 expression, TMB, defective mismatch repair (dMMR), or microsatellite instability (MSI+) as predictive biomarkers to guide clinical ICB therapy decisions.

The immune cytolytic activity (CYT) score is a new index of cancer immunity which implied the cytolytic T-cell activity [6]. We found CYT was significantly associated with MSI+ and TMB and may be an appealing, widely applicable indicator for the prediction of treatment response and clinical decision-making. An increased number of cytotoxic T lymphocytes (CTLs) at the invasive border has been demonstrated to be a reliable independent predictor of survival in patients with endometrial cancer [7].

However, less is known on the clinical and genomic features associated with CYT and its interplay with cancer cells in immune microenvironment (TME) of endometrial cancer patients. Filling this information gap will improve our understanding of molecular mechanisms that modulate immune surveillance and aid in the development of new therapeutic strategies for endometrial cancer patients.

## 2. Materials and Methods

### 2.1. Tumor Datasets

Clinical information was collected from the TCGA's public access pan-cancer project, which is strongly recommended for clinical elements and survival outcome analysis first. “Level 3” gene expression data, mutational annotation format (MAF) files, and copy number variation (CNV) files were all retrieved from TCGA database.

### 2.2. Determination of the Immune Cytolytic Activity

We obtained fragments per kilobase million (FPKM) values of endometrial samples from the TCGA-UCEC dataset. The FPKM values were transformed into transcript per million (TPM) values with following formula:

(1) $$\begin{array}{c} \text{TPM}=\frac{{\text{FPKM}}_{i}}{\sum_{i=1}^{n}{\text{FPKM}}_{i}}. \end{array}$$

We calculated each patient's immune CYT level, according to the expression of GZMA and PRF1 in TPM after adding a 0.01 offset to remove the zero from the equation.

The cutoff, median CYT value, was used to divide cancer patients into two immunological cytolytic groupings (CYT-high and CYT-low categories).

### 2.3. Prognosis of CYT-High and CYT-Low Subsets

The Kaplan–Meier method with log-rank test and/or univariable Cox regression analysis were used to compare prognosis between two groups and amongst tumor subtypes in overall survival (OS), disease-free survival (DDS), progression free interval (PFI), and disease-free interval (DFI) through survival and survminer (*R* package version 3.2–13 and 0.4.9) after filtering the indicating period shorter than 30 days. A *p* value less than 0.05 was considered as statistically significant.

### 2.4. Predication of Tumor Infiltrating Immunity

The CIBERSORT [8] was used to quantify the immune infiltration abundance of 22 types of immune cells in EC tumor samples. ESTIMSTE [9] was used to predict the ESTIMSTE score (purity), immune score, and stromal score in TME. We collected data from TCIA [10] for prediction of response to CTLA-4 and anti-PD-1 antibodies. The TIS score was calculated as an average value of log2-scale normalized expression of the 18 signature genes [11, 12].

### 2.5. Determination of Tumor Mutations, Clonal, and Neoantigens

The TCGA database was used to obtain EC's somatic mutation data. Somatic mutation information is available for 484 patients. TMB, which is defined as the number of somatic mutations per mega base of probed genomic sequence, differs amongst cancers. The total number of somatic nonsynonymous point mutations within each sample and TMB was calculated using the maftools [13] (*R* package version 2.10.0). TCIA provided us with information on the cellular composition of cancer neoantigens per Mb in each UCEC's two cytolytic subgroups [10]. The information including quantity of neoantigens, quantity of clonal/sub-clonal neoantigens were retrieved from TCIA [10] for patients with UCEC. The information included SNV neoantigens, Indel neoantigens, TCR/BRC richness, TCR/BRC Shannon, intratumor heterogeneity, and nonsilent/silent mutation rate were retrieved from TCGA Pan-Cancer Atlas for patients with UCEC.

### 2.6. Gene Enrichment Analysis

GSEA was performed with the ordered gene list which sorted by the Spearman's correlation coefficient between CYT score and other genes. The Molecular Signatures Database contains a collection of annotated gene sets for use with GSEA software (MSigDB [13]). The result of the GSEA shows the GO (biological process) enrichment and the normalized enrichment score (NES), which measures how overrepresented a gene set is at the top or bottom of a list of genes, and usually, absolute NES >1 and false discovery rate (FDR) less than 25% are held as significantly enriched in the Gene Set. GO (biological process) enrichment in SMGs of each CYT group was performed using clusterProfiler [14] and visualized with GO plot [15] (*R* package 1.10.2).

### 2.7. SMGs and CNV Analyses in UCEC

GISTIC2 [16] was used to analyze each dataset's tumors and compare copy number variations between the two cytolytic subtypes. Maftools was used to visualized the results for BubblePlot and ChromPlot, with a default FDR cutoff 0.1.

MutSig [17] was used to discovery the most significant genes of each CYT subgroup with FDR <0.1. Co-mutation plot was plotted with maftools. The correlation between SMGs and CYT in each CYT subgroup was calculated with Spearman's rank correlation.

### 2.8. Tumor Heterogeneity within Cytolytic Subsets

By clustering the variant allele frequencies as indicated by the width of their distribution, we were able to infer intratumoral genomic heterogeneity. With default options, efficient analysis, visualization, and summarizing of (MAF) files from large-scale cohort-based cancer studies, maftools assigned a mutant-allele tumor heterogeneity (MATH) score to each tumor sample.

### 2.9. Statistical Analyses

All statistical analyses were carried out in *R* (https://www.r-project.org/, version 4.12), with the Mann–Whitney *U* test to analyze the differences between two groups for continuous variables. The Spearman's rank correlation test was used to examine the correlation between variables. A two-sided *p* < 0.05 was considered statistically significant in all analyses.

## 3. Results

### 3.1. High CYT in EC Was Significantly Associated with Better Prognosis

**Figure 1:**
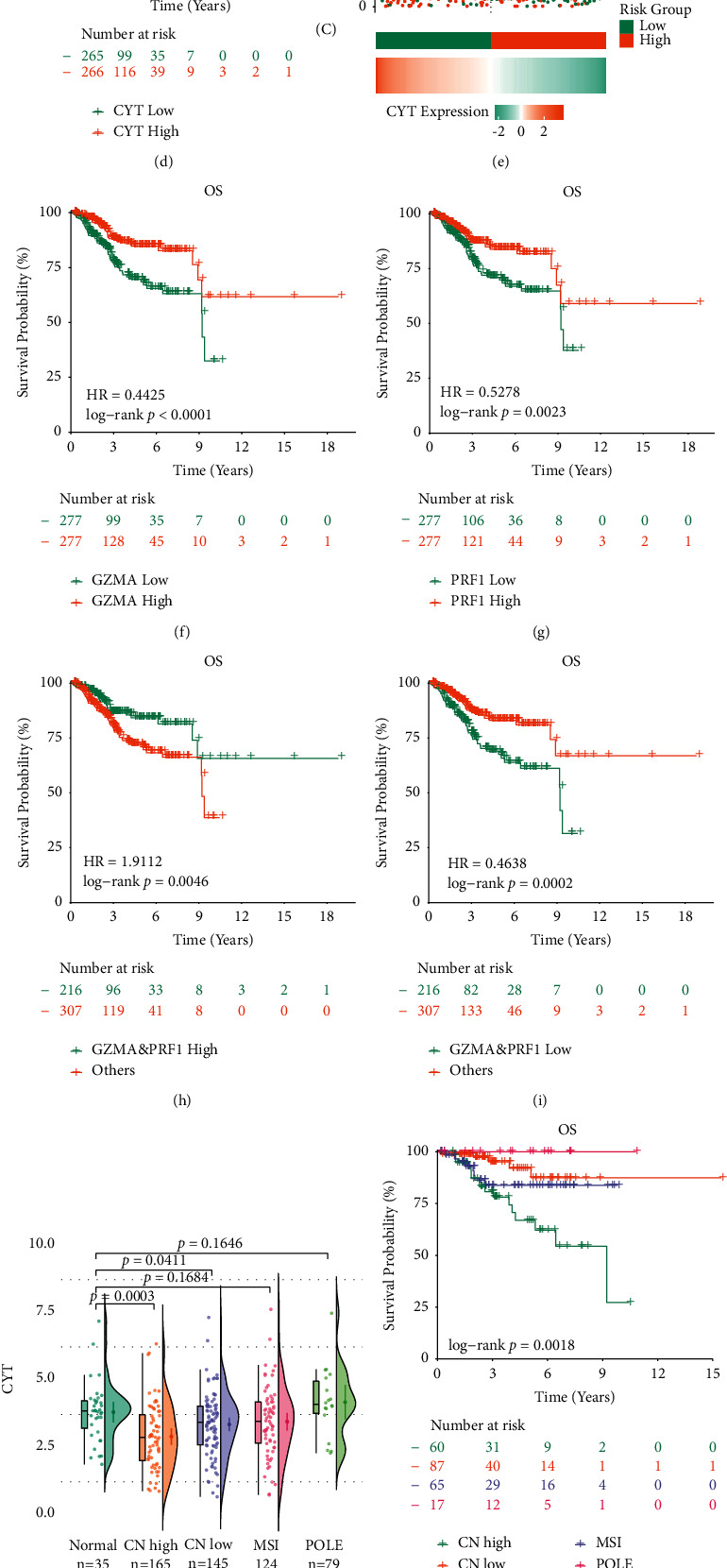
Association of the CYT with clinical features in EC. (a) CYT levels were lower in the UCEC datasets in contrast with the normal endometrium (tumor *n* = 551, normal *n* = 35); (b) paired samples between EC and normal tissue (*n* = 23); (c) both GZMA and PRF1 protein level were weakly expressed or not expressed in IHC protein expression data from the human protein atlas (HPA); (d) Kaplan–Meier method with log-rank test and univariable cox regression analysis were utilized to compare survival curves between two CYT groups in OS, HR (hazard ratio); (e) the curve of risk score, survival status of the patients, and heat map of CYT level were shown; (f–i) Kaplan–Meier curve evaluation of GZMA and PRF1 indicated that high level of both GZMA and PRF1 in EC synergistically affected the patients' overall survival; (j) CYT expression tended to be lower in CN high and CN-low UCEC subtypes but higher in POLE subtype when compared with normal tissue; (k) KM survival curves of each molecular subtype EC; and (l-m) CYT levels were significantly lower in ESC histologic subtype which possessed a shorter survival time probability.

The level of cytolytic activity was relatively lower in endometrial cancer versus normal endometrium (Figures 1(a) and 1(b), *p*=0.041, *p*=0.035). GZMA was shown to be lowly expressed in only 1/12 endometrial malignant samples and undetectable in 11/12 of those, according to HPA-derived protein expression data. Similarly, the negative expression of PRF1 was detected in 11 endometrial carcinoma samples (Figure 1(c)). Correlation analyses were conducted between CYT score and T cell receptors as well as ligands in UCEC patients, which was shown that the CYT score had a considerably positive correlation with immunomodulation-related ligands (*p* < 0.0001, Figure S1A) and receptors (*p* < 0.0001, Figure S1B).

We further stratified EC data based on the CYT value, as “CYT-high” and “CYT-low” group, and utilized the log-rank test to determine the prognosis of EC. It indicated that patients with higher CYT score showed a prolonged OS (*p* = 0.0034), DSS (*p* = 0.0009), DFI (*p* = 0.0021), and PFI (*p* = 0.0002) (Figure 1(d), Figure S2A). To further explore the effect of CYT on endometrial cancer prognosis, we plotted a scatter plot of gene expression and the corresponding survival time in different samples (Figure 1(e)). High expression levels of both PRF1 and GZMA or alone were favorable to patients' OS. Inversely, cooccurring low-expressed PRF1 and GZMA resulted in the worst clinical outcome, implying the synergetic effect of both two genes on patients' survival in EC (Figures 1(f)–1(i)).

To explore the links between cytolytic T-cell activity and molecular subtypes in EC, we determined the variation of CYT values among four molecular subgroups of EC. POLE subtype accounts for approximately 7% in EC [18] and had the highest CYT value and best clinical outcomes amongst the four molecular subtypes. MSI and CN-low subtypes accounts for around 28% and 39% in EC, respectively, with a moderate CYT levels and prognosis amongst the four molecular subtype EC, and CN High subtype, accounting for almost 26% in EC, owning the lowest CYT level (*p*=0.0003) and worst prognosis (Figures 1(j) and 1(k) and Figure S2C).

In TCGA-UCEC dataset, EC was classified into three histological subtypes, endometrioid endometrial adenocarcinoma (EEC), serous endometrial adenocarcinoma (ESC), and mixed serous and endometrioid (mixed). To investigate the connection between endometrial cancer histological subtypes and CYT levels, we displayed the dissimilarity of CYT value and prognosis between EEC and ESC histological ECs. ESC had the lower CYT values compared with normal endometrium and worse prognosis, and there was no obvious difference between EEC and normal endometrium (Figures 1(l) and 1(m) and Figure S2D).

Taken together, the high CYT level, which delegated aggressive the cytolytic T-cell activity, predicated the better clinical outcomes in endometrial carcinoma.

### 3.2. CYT Was Related to a Distinct Mutation Status in EC

**Figure 2:**
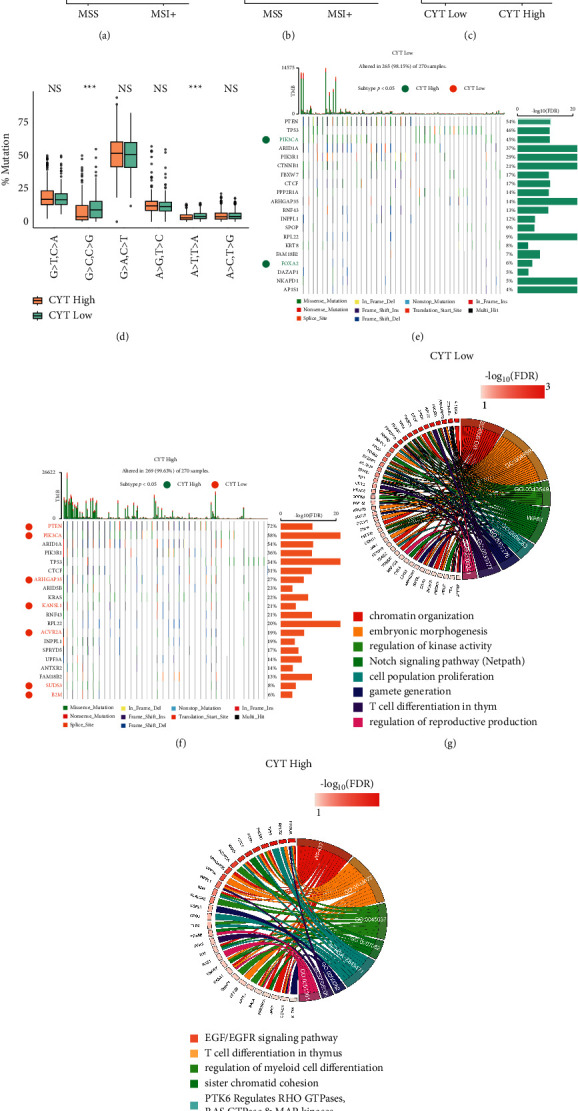
CYT correlated with discrete mutational events in EC. (a-b) TMB and CYT were higher in MSI + versus MSS tumors; (c) TMB was higher in CYT-high tumors; (d) transversion and transition mutations in CYT-high versus -low tumors; (e-f) oncoplot depicted SMGs (FDR < 0.1) in CYT-high versus -low tumors. The SMGs that correlate with the each cytolytic subtype (*p* < 0.05) are marked with green (CYT-low) or orange (CYT-high) circles besides its gene's name. The FDR for SMGs was plotted in −log10 on the right side of the plots; (g-h) GO BP enrichment chord plot for the SMGs.

Subsequently, we managed to determine whether CYT levels were correlated with distinct EC mutational signatures. In microsatellite instability (MSI+) ECs, the mutation load rose dramatically (*p* < 0.0001, Figure 2(a)). Consistent with previous findings in other cancers [19], also, the cytolytic activity was expected to be increased substantially in MSI + tumors (*p* < 0.0001, Figure 2(b)). Besides the mutation load increasing considerably in CYT-high tumors (Figures 2(a), 2(c), 2(d)), MSI + EC was associated with better clinical outcomes (Figure S2B).

Further analysis showed that most mutations across the dataset were related to *A* > *T* and *T* > *A* as well as *G* > *C* and *C* > *G* transversions, which were of higher percentage in CYT-low tumors, and other specific fragments did not differ between CYT-high and -low tumors (Figure 2(d)).

Next, we identified significant mutation genes (SMGs FDR < 0.1) by MutSig, CYT-low primary tumors were significantly associated with mutations in PIK3CA, FOXA2 (Figure 2(e)), whereas, CYT-high primary tumors with a totally different group of genes, including PTEN, PIK3CA, ARHGAP35, KANSL1, ACVR2A, SUDS3, and B2M (Figure 2(f)). The SMGs in each CYT subset enriched mainly in cell proliferation and T cell differentiation (Figures 2(g) and 2(h)).

### 3.3. Correlation of SCNA with the Cytolytic Activity in EC

**Figure 3:**
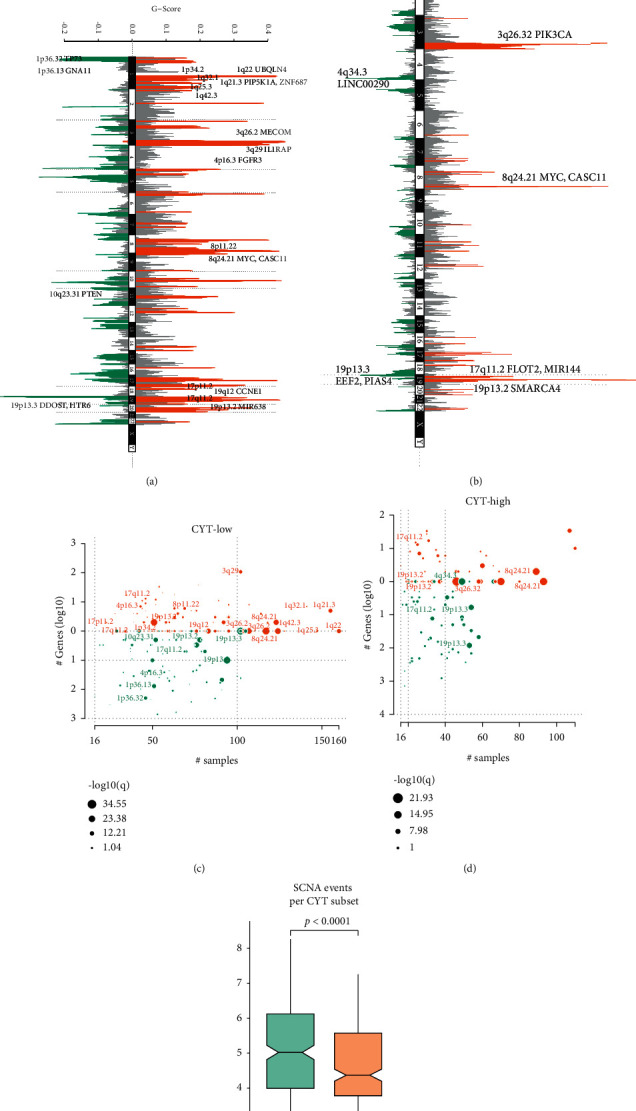
High cytolytic index was related to reduced CNV in EC. (a-d) Analysis of copy number data from TCGA-UCEC clusters using GISTIC 2.0 revealed significant amplifications (orange) and absences (green) of copy number. (e) In cytolytic-low tumors, total SCNA events score estimated for each EC patient were considerably higher.

Escalated genomic instability with broad somatic copy number alterations (SCNA) is the characteristic in EC patients [20]. We then conducted the GISTIC2 [16] analysis to evaluate the correlation of copy number variations (CNV) with cytolytic activity in different CYT subtypes. First, we compared the copy number alteration between CYT-low and -high tumors. As shown in Figure 3, amplification of 8q24.21 (*MYC, CASC11*), 19q12 (*CCNE1*), 4p16.3 (*FGFR3*), 3q29 (*IL1RAP*), 1q21.3 (*PIP5K1A, ZNF687*), etc. and deletion of 10q23.31 (*PTEN*), 1p36.32 (*TP73*), 1p36.13 (*GNA11*), and 19p13.3 (*DDOST, HTR6*) were more frequently detected in CYT-low tumors (Figure 3(a)). While amplification of 8q24.21 (*MYC, CASC11*), 3q26.32 (*PIK3CA*), 17q11.2 (*FLOT2, MIR144*), 19p13.2 (*SMARCA4*), and deletion of 4q34.3 (*LINC00290*), 19p13.3 (*EEF2, PIAS4*) were more frequent shown in CYT-high EC (Figure 3(b)). Although some SCNA events coincided in both CYT-high and -low tumors, CYT-high EC displayed even lower *G*-scores in corresponding loci, such as 3q26.2 and 1q21.3. Altogether, CYT-high tumors harbored significantly fewer SCNA events than cytolytic-low tumors (*p* < 0.0001, Figure 3(e)).

### 3.4. Association between CYT and Enrichment of Tumor Neoepitope

**Figure 4:**
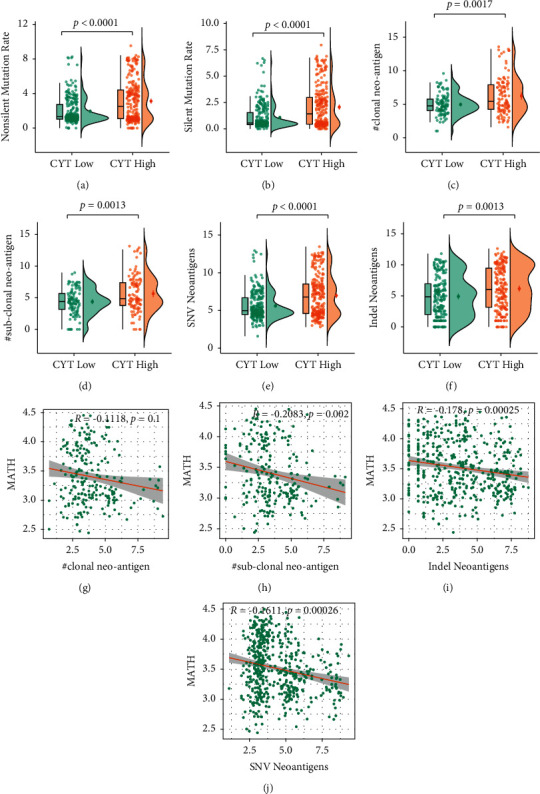
Association between the CYT and neoepitope load. (a-f) Raincloud plots of the silent and non-silent mutation rate, number of clonal and sub-clonal neoantigens, single-nucleotide variants (SNV) and indel neoantigens by low and high CYT score groups. (g-j) Spearman correlation analysis was performed to explore the relationship between clonal/sub-clonal/SNV/indel neoantigens and MATH scores.

Neoepitopes are derived from peptides encoded by tumor somatic mutations and are hence not subject to central tolerance in the thymus. It has been shown to be preferentially recognized by immune system and induce antitumor T-cell activation [21, 22]. Therefore, we further analyzed whether the mutational/neoantigen load was distinctive in EC patients with regard to CYT status. As shown in Figure 2(c), a substantially increased TMB was detected in CYT-high EC (*p* < 0.0001, Figure 2(c)). Accordingly, both the silent mutation and nonsilent mutation rate were shown to be higher in CYT-high ECs (*p* < 0.0001, Figure 4(a) and 4(b)).

To investigate the linkage between CYT and the clonal/sub-clonal neoantigen burden, the predicted neoantigens and the clonal/sub-clonal categorization for each neoantigen were derived from the TCIA database [10]. Consistent with the TMB pattern, significant increase of neoantigens/sub-neoantigens (*p*=0.0017, *p*=0.0013, Figures 4(c) and 4(d)) and Indel/SNV neoantigens (*p* < 0.0001, *p*=0.0013, Figures 4(e) and 4(f)) were exhibited in CYT-high EC. However, the MATH score did not correlate (absolute *R* < 0.3) with the number of neoantigens/sub-neoantigens and Indel/SNV neoantigens in EC (Figures 4(g)–4(j)), which suggested that CYT-high tumor had more neoantigens not owing to tumor heterogeneity. Taken together, these data suggested that cytolytic activity in EC might be driven by elevated mutation and/or neoepitope load.

### 3.5. CYT-High EC Correlated to Essentially Higher Levels of Apolipoprotein B

**Figure 5:**
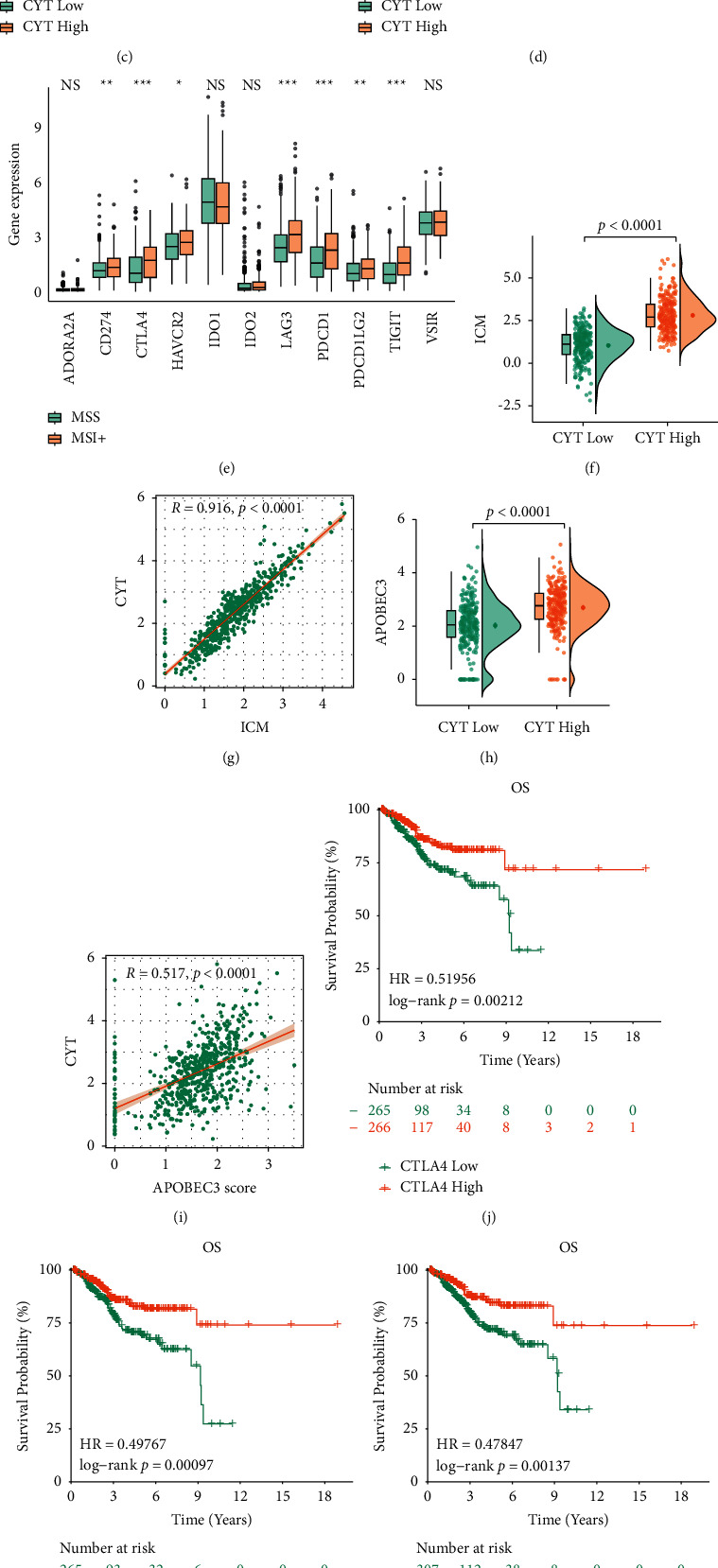
Expression of apolipoprotein B cytokine and ICMs in CYT subgroups. (a, h) Individual variation in APOBEC3 gene expression between CYT groups and combined APOBEC3 score; (b-d, f) cytokine, treg markers, individual ICMs and ICM score differed in CYT-high and -low EC; (e) contrast of individual ICMs between CYT-high and -low EC; (g, i) ICM and APOBEC3 scores were positively correlated with CYT values. (j–m) After a synergistic analysis for CTLA-4 and PD-L1, Kaplan–Meier curves depicted the overall survival of EC patients.

We speculated that gene expressions of APOBEC3 (apolipoprotein B mRNA editing catalyzed polypeptide 3) members were enhanced in CYT-high EC, resulting in an increased mutation load in the TME (Tumor microenvironment), as the cytolytic activity was positively correlated with a neoantigen load and the mutation load in EC. Based on this notion, we investigated the relationship of APOBEC3 mutation status with CYT status in EC. As displayed in Figure 5, CYT-high EC was linked to a vastly higher APOBEC3 family member expression (Figure 5(a)). The geometric average expression of the following genes [23] was used to calculate the APOBEC3 score: APOBEC3A, APOBEC3B, APOBEC3C, APOBEC3D, APOBEC3F, APOBEC3G, and APOBEC3H. This score, which reflects the overall amount of apolipoprotein B family activity, was similarly higher in CYT-high tumors (*p* < 0.0001) and was strongly associated with the CYT score (*R* = 0.517, *p* < 0.0001) (Figures 5(h) and 5(i)).

### 3.6. ECs with High and Low Cytolytic Activity Had Varied Cytokine Expression Patterns of ICM

Balli [24] and Zaravinos et al. [19] recently reported increased expression of pro- and anti-inflammatory cytokines and immune checkpoint molecules in CYT-rich tumors. Also, it has been reported that for patients with high PD-L1 expression in tumor cells, PD-1/PD-L1 blockade is more effective than in those with a low expression of PD-L1 [25–27], which may be due to the sensitivity to immune checkpoint with anti-PD-1 treatment. Therefore, we speculated that the expression of cytokines and chemokines was expected to be significantly increased in endometrial tumors with high CYT (Figure 5(b)). We found that several ICM (immune checkpoint molecules) were highly expressed in endometrial carcinomas with high MSI and CYT (Figures 5(b)). In particular, PD-1, CD274 (PD-L1), PD-L2, CTLA4, indolamine 1 (IDO1), lymphocyte activation gene 3 (LAG3), VSIR, ADORA2A, HAVCR2, and T immunoreceptors with Ig and ITIM domains (TIGIT) showed marked overexpression in tumors with elevated CYT, whereas the varied IDO2 expression was negligible between high and low CYT tumors (Figure 5(d)). Moreover, we also calculated the expression of twelve known immune checkpoint molecules in MSI+ and MSS tumors and found significantly higher expression of many genes (except for ADORA2A, IDO1/2 and VSIR) in MSI ECs (Figure 5(e)).

In addition, we determined that in ECs, both individual and high levels of CTLA-4 and PD-L1 synergistically had a positive impact on the patients' OS (Figures 5(j)–5(l)). Conversely, the low expression of CTLA-4 and PD-L1 together contributed to the opposite outcome (Figure 5(m)). These results provided evidence that high expression of both immune checkpoints affected synergistically the survival of endometrial cancer patients.

Furthermore, we also found higher level of cytokines and chemokines in CYT-high ECs, and a solid relationship between CYT and the ICM index utilizing six gene expressions model (Figures 5(f) and 5(g)), which suggested that CYT could reflect ICM expression. Treg markers, such as FOXP3, CCR4, CCR5, and IL2RA, were at a high level expression in CYT-high EC (Figure 5(c)).

In summary, in endometrial cancer with MSI and high CYT, immune escapement might be regulated by the overexpression of diverse immune checkpoint genes, expediting tumor cells with selective stress to elude a cytotoxic T cell immune response.

### 3.7. High CYT EC Remarkably Enriched a Favorable Immune-Related Gene Set

**Figure 6:**
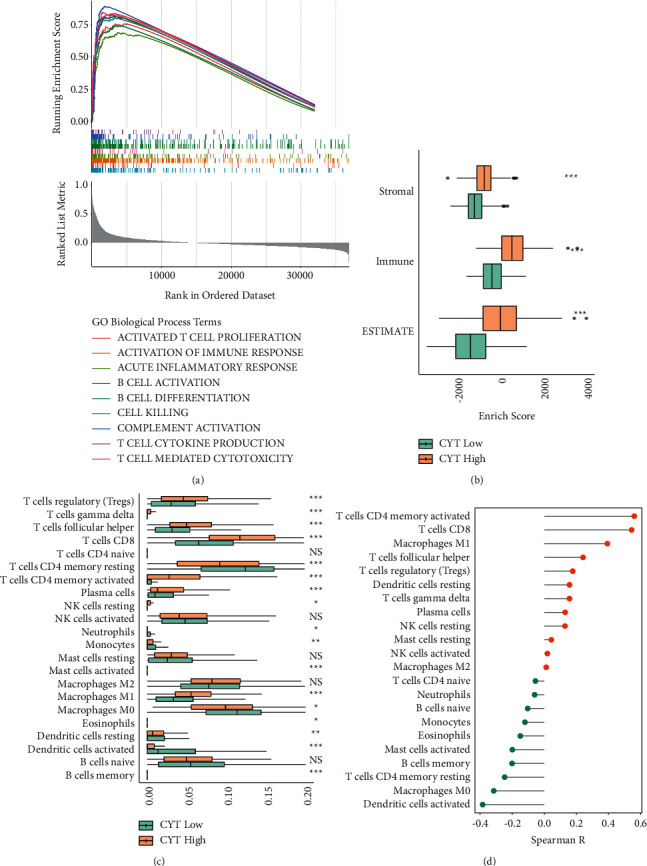
Immune activity in CYT subsets (a) enrichment plots are displayed. For all enrichment terms, absolute NES > 1 and FDR < 0.25; (b) in the CYT high and low subsets, the boxplot displayed ESTIMATE score, immune score, and stromal score; (c) the boxplot exhibited the difference between immune cell infiltration level between CYT-low and high subgroup; and (d) CYT score was correlated to various immune cells.

It was expected that EC with a high CYT would have a favorable TME as it was related to better survival. Therefore, the GO biological process (BP) GSEA was performed in tumors with disparate CYT levels. As displayed in Figure 6, high CYT EC essentially enriched numerous immune-promoting GO gene terms, such as activated T cell proliferation, T cell mediated cytotoxicity, cell killing, and B cell activation (Figure 6(a)).

### 3.8. Composition of Immune Activating and Suppressing Cells in CYT-High EC

As the high CYT UCEC immune-related gene clusters enriched, it was necessary to identify the composition of immune cells in the malignancies. Through CIBERSORT result, anticancer immune cells, such as activated CD8^+^*T* and CD4^+^ memory T cells, M1 Macrophages were identified to be markedly infiltrated in the high CYT-high EC and were substantially positively correlated with CYT (*p* < 0.001 and *R* > 0.3). While activated dendritic cells (*p* < 0.001) and M0 macrophages (*p* < 0.05) were abounded in CYT-low group and negatively connected with the CYT score (*R* < −0.3) (Figure 6(c) and 6(d)).

**Figure 7:**
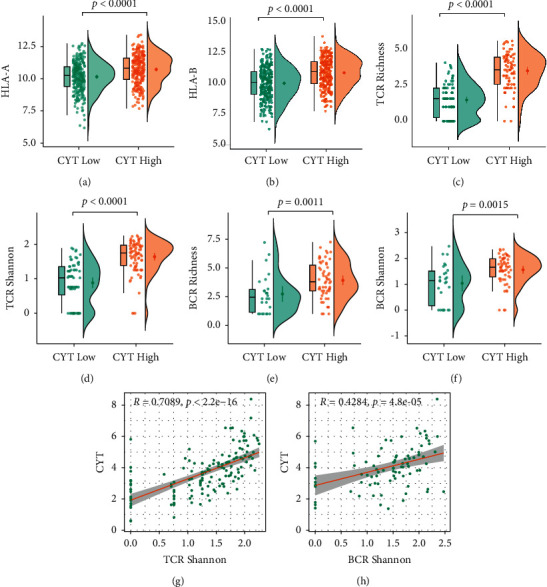
Association between CYT and TIL receptor. (a–f) HLA-A, HLA-B, TCR/BCR richness and Shannon were all higher in CYT-high ECs; (h-i) CYT score and TCR Shannon/richness had a strong relationship.

However, there was an abundance of immune inhibitory cells, such as Treg cells (Figures 6(c) and 6(d)) in CYT-high EC. Furthermore, the ESTIMATE algorithm was also applied to determine the tumor purity and immunity in TME. As the ESTIMATE score was negatively correlated with tumor purity, tumor purity was lower in CYT-high group while immune and stromal scores were higher (Figure 6(b)). Importantly, HLA-A and HLA-B, two vital molecules of the major histocompatibility complex (MHC), had a significantly higher expression in CYT-high EC (*p* < 0.0001, Figures 7(a) and 7(b)).

As antigen-specific TCR and BCR were also an important feature of the immune system for recognizing pathogens and cancer cells. From the analysis, we further found that significantly increased TCR/BCR richness (Figures 7(c), 7(e)) and TCR/BCR Shannon (Figures 7(d), 7(f)–7(h)), which represented the diversity of TCR and BCR and possibly their improved anticancer efficacy was associated with CYT-high EC.

These results indicated that, compared with tumors with low CYT, tumors with high CYT were associated with an enhanced immune response and favorable anticancer immune cells.

### 3.9. The CYT-High Tumors Responded Effectively to ICB Therapy

**Figure 8:**
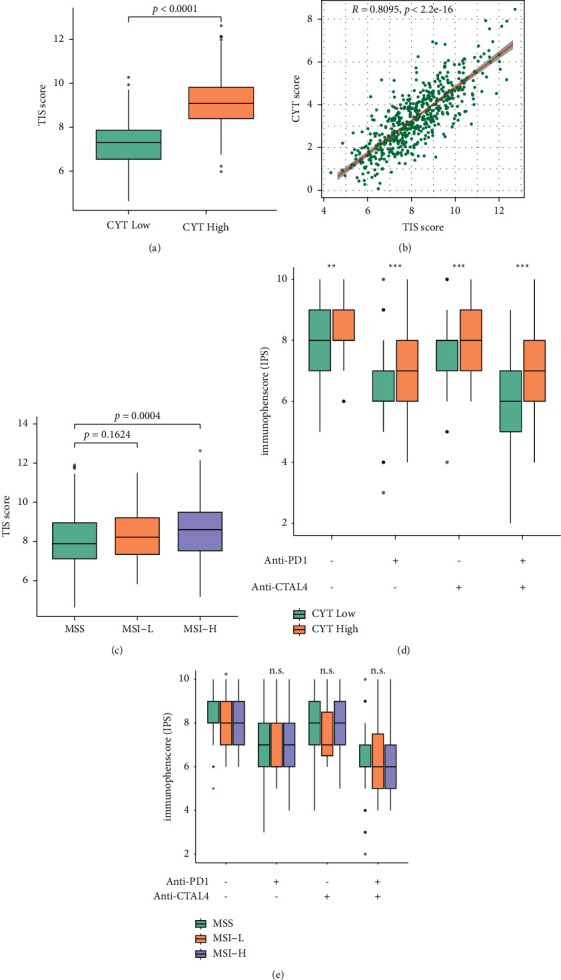
Prediction of EC patients' response to immune checkpoint inhibition. (a, c) Tumor inflammation signature (TIS) scores differed in CYT subsets and MSI groups; (b) correlation between CYT score and TIS score; (d-e) IPS scores across the CYT and MSI subgroups, respectively.

CYT-high ECs were characterized by an escalated expression of the human leukocyte antigen class II (HLA-II) complex and genes involved in the antigen presentation pathway. Also, the high levels of the therapeutic targets PD-L1 and CTLA4 in CYT-high EC patients implied that they could be treated with immune checkpoint inhibitors. Supporting this hypothesis, all samples belonging to high CYT score in the tumor inflammation signature (TIS) score were reported to be correlated with a response to anti PD-L1 checkpoint inhibitor pembrolizumab (*p* < 0.0001, Figure 8(a)). We also noticed that the CYT score had a correlation with TIS score (*R* = 0.8095, *p* < 0.0001, Figure 8(b)). According to the previous report, MSI + tumors might have high possibility to response to ICB therapy, but the TIS score was higher only in MSI-H EC when compared with MSS EC (*p* = 0.0004, Figure 8(c)).

Immunophenoscore (IPS) could be a learning-based scoring machine model that might help to in bulk sequencing data make predictions of patients' response to ICB. Two subtypes of IPS values could function as the indicators of response to anti-PD-1/PD-L1 and anti-CTLA-4 therapy. In this way, CYT-high patients were more likely to respond to ICB therapy (Figure 8(d)). While diversified MSI status EC group presented no significant variation of IPS (Figure 8(e)). These data indicated that inferring patients with high CYT might be suitable candidates for ICB therapy. And high CYT score, might worked as an indicator of ICB therapy, facilitating favorable immunophenotype.

## 4. Discussion

The discoveries and successful clinical implications of immune checkpoint inhibitors (ICI) have revolutionized treatment regiments for cancer patients, particularly in advanced-stage patients with various solid cancer, and extremely improved the clinical outcome. Currently, limited biomarkers can be used to stratify patients for ICI immunotherapies, including expression levels of PD-L1, tumor mutation burden (TMB), and status of microsatellite instability (MSI). However, defined biomarkers to guide effective stratification of cancer patients for using immune checkpoint inhibitors are controversial and remain to be determined. Increasing evidence has indicated that exploration of publicly genomic database by bioinformatic tools would discover comprehensive biomarkers for more accurate stratification of cancer patients for ICIs. Thus, in this study, we sought to conduct an extended comprehensive analysis of the EC transcriptional and genetic landscape to identify and confirm the value of cytolytic immune activity for clinical application of ICI in EC patients. Through stratifying EC patients with a cytolytic gene expression signature, we distinguished a subset of them with striking T-cell reactivity. First, we elucidated the clinical properties associated with CYT levels in EC and we found that the CYT value was lower in endometrial cancer than that in normal endometrium. Among four molecular subtypes of EC, the POLE subtype harbored the highest CYT levels with the best prognosis, while the CN high molecular subtype had the lowest CYT levels and the worst prognosis. Similarly, ESC had a lower CYT value and worse prognosis compared to EEC in terms of histological subtypes. These results implied that the CYT value was closely associated with clinical outcomes and would be a predictor for ICI and prognosis in EC.

To further determine the correlation of neoepitope load with cytolytic activity and mutation burden in EC, we performed a comprehensive analysis of the initial characterization of neoepitope load in cancers through mining large-scale TCGA dataset. We found that high cytolytic activity was closely related to mutation load and predictive neoepitopes for EC. This finding was also in accordance with the correlation between cytolytic index and mutational burden in other certain tumors such as lung and stomach adenocarcinoma [6], where anti-PD-1 antibodies displayed significant clinical tumor regression. Indeed, several studies have shed light on the powerful potentialities of tumor neoepitopes in T-cell recognition of tumor cells, raising the attention in patient-specific immunotherapeutic strategies on cancer treatment such as personalized vaccines [28, 29]. As a result, the tight association of neoepitope load with the cytolytic activity index in EC could reflect the antitumor activities which was analogous to that present in other ICI-sensitive tumors, indicating that neoepitope load may also be the promising predictor to determine ICI sensitivity in EC [22, 30].

In addition to strong correlation of cytolytic activity with transcriptional and genetic subtypes of the cancer, further analysis showed that EC with low CYT tended to be harbored accelerated genomic structural variations, particularly *MYC*, *CCNE1,* and *FGFR3* amplifications and/or deletions of *PTEN* and *TP73* tumor suppressor genes. Similarly, data from mouse tumor models with hepatocellular carcinoma and melanoma have revealed that *MYC* amplification was correlated with low CYT and few T-cell infiltration. These findings suggested that, in addition to neoepitopes and genomic structural variations, especially CNVs or other complex structural variations such as chromothripsis and/or chromoplexy may promote the generation of immunosuppressive microenvironment as well [31, 32]. Consequently, the co-evolution of genomic alterations in tumor cells with the stromal components in EC not only accelerated tumor progression but also reinforced the resistance to drug treatment including immunotherapy with ICIs.

Importantly, our data showed that CYT-high endometrial tumors had a considerably higher TIL density, which was linked to better overall survival. These findings were also in accordance with several recent studies that increased immunity, and cytolytic activity of T cells and M1 macrophages were thought to be related with prolonged survival in EC. Furthermore, we found that higher CYT levels were associated with the overexpression of multiple immune-checkpoint molecules, such as PD-1, PD-L1, CTLA4, LAG3, TIGIT, and VSIR, supporting that in addition to PD-1, PD-L1, and CTLA4, more immune checkpoint molecules could be as promising targets for immunotherapy. Finally, MSI + EC patients with elevated cytolytic levels were likely to have generated a strong immune response in response to the neoepitope. However, some MSI + CYT-high EC patients showed ineffective responses to ICIs due to increased levels of various immunosuppressive checkpoint molecules with complex genome alterations. The heterogeneity of immune reactions in EC also suggested that comprehensive genomic profiling (CGP) with a deep analysis of data would be powerful tools for precision treatment decision, and targeting multiple immune checkpoint pathways simultaneously or in combination with other therapeutic strategies such as the targeting therapy would be advantageous for patients with endometrial carcinoma.

## 5. Conclusion

In summary, our data supported the notion and possibility that integrated and comprehensive analysis of genomic and immunophenotypic data would be helpful to understand immunostimulant and reactions in EC, and CYT value was likely to be a promising predictor for response to immunotherapy with ICIs and prognosis of EC patients. It was also worth considering that comprehensive genomic profiling (CGP) with CYT evaluation may exert the additional value for guiding effective immunotherapy or in combination with other therapeutic regiments in EC, particularly refractory EC patients. Finally, it was reasonable to believe that the notion of CYT evaluation through genomic analysis would move beyond the standard stratification of EC in currently clinical practice and provide new evidence to predict T-cell immunity in EC similar to that in melanoma, colon, and lung cancer [19, 33–34], which will be more beneficial for patient management.

## Data Availability

The data used to support the findings of this study are available TCGA database: https://portal.gdc.cancer.gov, TCGA clinical data: https://api.gdc.cancer.gov/data/1b5f413e-a8d1-4d10-92eb-7c4ae739ed81, TCIA database: https://tcia.at/home, and HPA database: https://www.proteinatlas.org/.

## Additional Points

This article does not contain any studies with human or animal subjects directly, for all data were collected through public database.

## Supplementary Materials

Supplementary Materials Supplementary Figure 1: Association of CYT with CD8^+^ T cell ligand in EC. (A-B) The CYT score and CD8^+^ T cell ligands and receptors had a substantial connection including ligands (A) CD80, CD86, PD-L1, PD-L2, LGALS9, and TNFSF14; receptors (B) CTLA4, PD-1, TIM3, LAG3, BTLA, TIGIT. Supplementary Figure 2: Prognosis of differential CYT expression, MSI status, histological and molecular EC subtypes. (A) Survival curves between CYT-high and CYT-low tumors in DSS, PFI and DFI; (B) Survival curves between MSS and MSI tumors in OS, DSS, PFI and DFI; (C) Survival curves among EC molecular subtypes in DSS, PFI and DFI; (D) Survival curves between ECC and ESC histological tumors in DSS, PFI and DFI.
