# Supplementary figures and images for: Immune Cytolytic Activity for Comprehensive Insights of the Immune Landscape in Endometrial Carcinoma
Source: J Oncol. 2022 Jul 18;2022:9060243. doi: 10.1155/2022/9060243 (PMC9313908; doi:10.1155/2022/9060243)

**Figure S1**

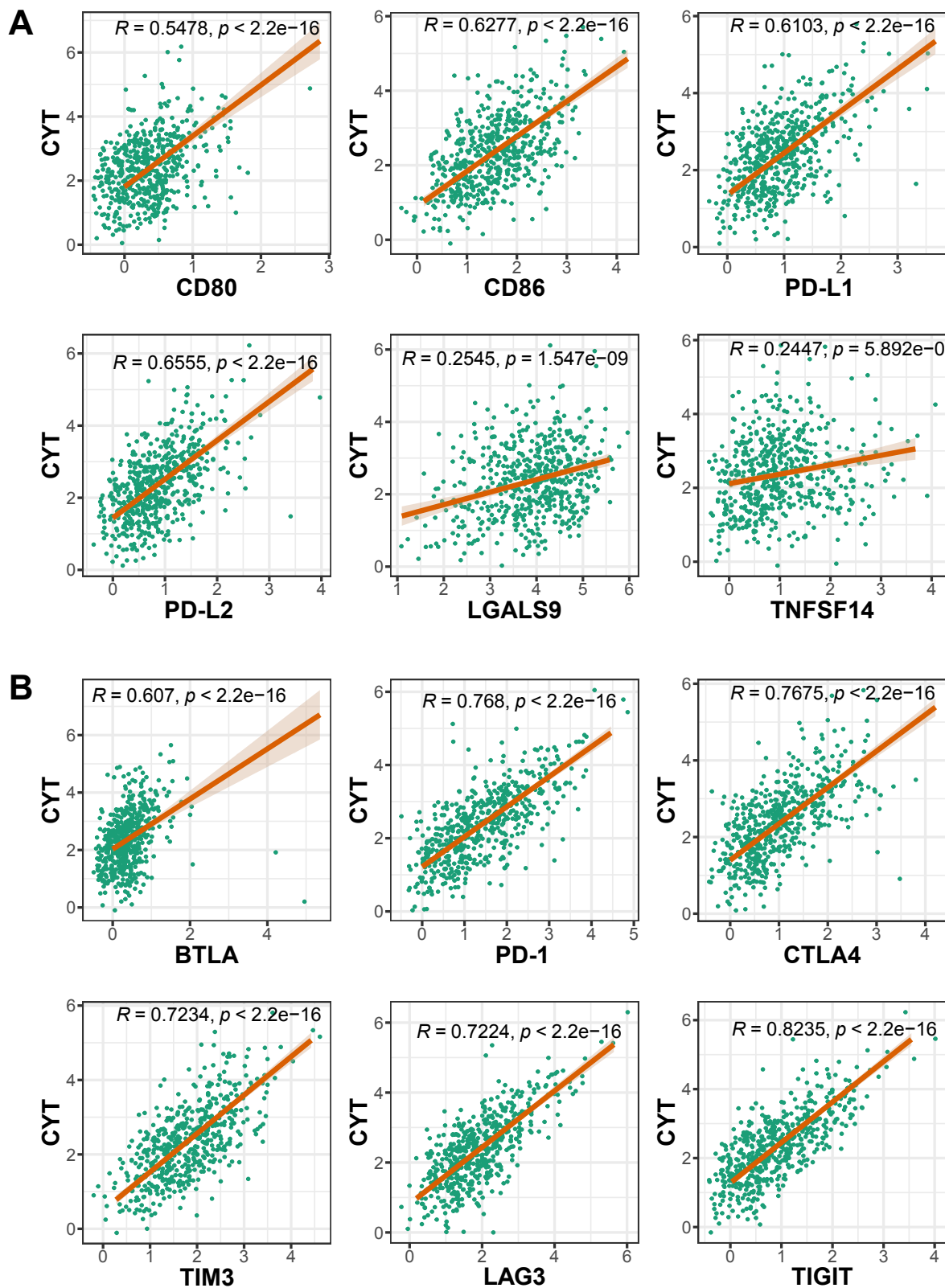

Supplement: Supplementary Materials — Supplementary Figure 1: Association of CYT with CD8+ T cell ligand in EC. (A-B) The CYT score and CD8+ T cell ligands and receptors had a substantial connection including ligands (A) CD80, CD86, PD-L1, PD-L2, LGALS9, and TNFSF14; receptors (B) CTLA4, PD-1, TIM3, LAG3, BTLA, TIGIT. Supplementary Figure 2: Prognosis of differential CYT expression, MSI status, histological and molecular EC subtypes. (A) Survival curves between CYT-high and CYT-low tumors in DSS, PFI and DFI; (B) Survival curves between MSS and MSI tumors in OS, DSS, PFI and DFI; (C) Survival curves among EC molecular subtypes in DSS, PFI and DFI; (D) Survival curves between ECC and ESC histological tumors in DSS, PFI and DFI. [file 9060243.f1.zip › 9060243.f1/FS1 (1).pdf]

Figure S2

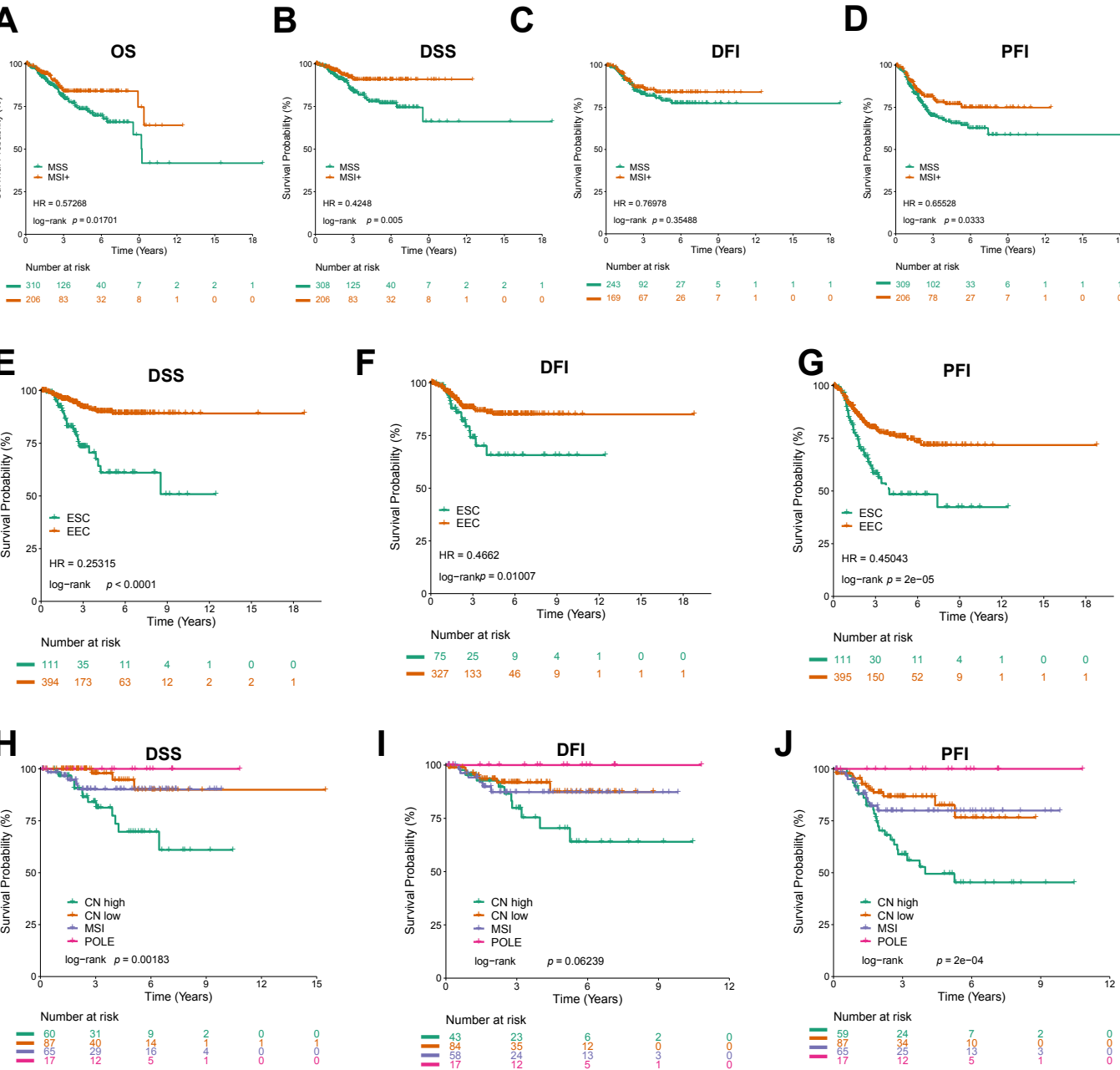

Supplement: Supplementary Materials — Supplementary Figure 1: Association of CYT with CD8+ T cell ligand in EC. (A-B) The CYT score and CD8+ T cell ligands and receptors had a substantial connection including ligands (A) CD80, CD86, PD-L1, PD-L2, LGALS9, and TNFSF14; receptors (B) CTLA4, PD-1, TIM3, LAG3, BTLA, TIGIT. Supplementary Figure 2: Prognosis of differential CYT expression, MSI status, histological and molecular EC subtypes. (A) Survival curves between CYT-high and CYT-low tumors in DSS, PFI and DFI; (B) Survival curves between MSS and MSI tumors in OS, DSS, PFI and DFI; (C) Survival curves among EC molecular subtypes in DSS, PFI and DFI; (D) Survival curves between ECC and ESC histological tumors in DSS, PFI and DFI. [file 9060243.f1.zip › 9060243.f1/FS2 (1).pdf]
